# Short- and long-run goals in ultimatum bargaining: impatience predicts spite-based behavior

**DOI:** 10.3389/fnbeh.2015.00214

**Published:** 2015-08-17

**Authors:** Antonio M. Espín, Filippos Exadaktylos, Benedikt Herrmann, Pablo Brañas-Garza

**Affiliations:** ^1^Department of Economics, Business School, Middlesex University LondonLondon, UK; ^2^GLOBE, Departamento de Teoría e Historia Económica, Universidad de GranadaGranada, Spain; ^3^BİLGİ Economics Lab of IstanbulIstanbul, Turkey; ^4^School of Economics, University of NottinghamNottingham, UK

**Keywords:** ultimatum game, costly punishment, delay discounting, impatience, fairness, competitive spite

## Abstract

The ultimatum game (UG) is widely used to study human bargaining behavior and fairness norms. In this game, two players have to agree on how to split a sum of money. The proposer makes an offer, which the responder can accept or reject. If the responder rejects, neither player gets anything. The prevailing view is that, beyond self-interest, the desire to equalize both players’ payoffs (i.e., fairness) is the crucial motivation in the UG. Based on this view, previous research suggests that fairness is a short-run oriented motive that conflicts with the long-run goal of self-interest. However, competitive spite, which reflects an antisocial (not norm-based) desire to minimize others’ payoffs, can also account for the behavior observed in the UG, and has been linked to short-run, present-oriented aspirations as well. In this paper, we explore the relationship between individuals’ intertemporal preferences and their behavior in a citywide dual-role UG experiment (*N* = 713). We find that impatience (short-run orientation) predicts the rejection of low, “unfair” offers as responder and the proposal of low, “unfair” offers as proposer, which is consistent with spitefulness but inconsistent with fairness motivations. This behavior systematically reduces the payoffs of those who interact with impatient individuals. Thus, impatient individuals appear to be keen to minimize their partners’ share of the pie, even at the risk of destroying it. These findings indicate that competitively reducing other’s payoffs, rather than fairness (or self-interest), is the short-run goal in ultimatum bargaining.

## Introduction

The ultimatum game (UG) is an economic experimental set-up widely used to study the nature of human bargaining and the enforcement of fairness norms (Güth et al., 1982; Sanfey et al., 2003; Henrich et al., 2005, 2006). In this game, the first player (the proposer) proposes how to split a sum of money with the second player (the responder). The responder can either accept or reject the proposal. If the proposal is accepted, the “pie” is divided accordingly; if rejected, neither player is paid. Economic models based on narrow self-interest predict that the responder should accept any positive offer, at least in non-repeated interactions. By backward induction, the proposer should offer the smallest positive amount to the responder, and the latter will accept the deal. Assuming money-maximizing players, these patterns of behavior constitute the subgame perfect equilibrium of the one-shot UG. However, experimental evidence has consistently contradicted these predictions as responders very often reject “unfair,” albeit positive offers and most proposers offer “fair,” equal splits (Camerer, 2003).

Explaining proposers’ generous offers is straightforward from a strategic viewpoint: since a low offer will likely be rejected, it is in the proposer’s self-interest to make a high offer to avoid coming out empty-handed (Roth et al., 1991; Wells and Rand, 2013). Strategic reasoning does not apply, however, to responders’ observed behavior if future encounters with the same proposer are unlikely (otherwise, rejections might be used to encourage higher future offers). Most scholars have invoked normative, fairness-based rationales for the existence of rejections (and fair offers) in one-shot interactions: people dislike unfairness—defined in either simple forms like mere payoff inequality (Fehr and Schmidt, 1999; Bolton and Ockenfels, 2000) or more complex ones like intentional unfairness (“unkindness”) (Rabin, 1993; Charness and Rabin, 2002). In this vein, the rejection of a low offer is considered as an act of costly punishment implemented by fair-minded individuals.

Therefore, the mainstream view is that the decision making of both proposers and responders in the one-shot UG relies on a combination of (strategic) self-interest and fairness-based considerations. Less attention has been paid, however, to other motivations like (psychological) spite, the “ugly twin” of altruism, as a crucial force underlying observed behavior *in either role* of the UG. For a spiteful individual, other individuals are competitors whose payoffs negatively affect her own utility; that is, the welfare of other individuals enters negatively in her utility function (e.g., Kirchsteiger, 1994; Levine, 1998; see Fehr and Schmidt, 2006, for a review of other-regarding preferences models)^1^. Promoting the other party’s displeasure rather than one’s own benefit is indeed traditionally regarded as a fundamental goal in bargaining, very often leading negotiations to collapse (Loewenstein et al., 1989).

### Delay Discounting and Social Behavior

Recently, delay discounting (DD) is being used to assess the motivations behind social behavior (Curry et al., 2008; Crockett et al., 2010; Espín et al., 2012). DD measures individuals’ preferences for smaller-sooner over larger-later reward (see reviews in Frederick et al., 2002; Green and Myerson, 2004; Luhmann, 2009). Such intertemporal preferences are fairly stable within individuals across time (Kirby, 2009). If a specific choice involves trading off short-run and long-run incentives, those individuals who discount the future more heavily (i.e., short-run oriented, impatient individuals with a high rate of DD) tend to favor the former over the latter. Outside the arena of social behavior, there are many well-studied examples of how experimentally elicited DD predicts decision making in field situations that involve intertemporal trade-offs (Kirby et al., 1999; Chabris et al., 2008; Meier and Sprenger, 2012).

In the specific context of the one-shot UG, Crockett et al. (2010) found that higher DD (impatience) of responders, as measured by their choices in a standard task involving hypothetical monetary reward, predicts higher rejection rates. Under the traditional interpretation of the UG as a conflict between self-interest and fairness, this result suggests that short-run psychological incentives (costs or benefits), such as immediate negative emotions (Pillutla and Murnighan, 1996; Sanfey et al., 2003; Van’t Wout et al., 2006) or psychological satisfaction (de Quervain et al., 2004), underlie the decision to impose fairness at a personal cost. Accordingly, promoting long-run material self-interest would require overriding the immediate, very likely emotional, impulse to punish violations of fairness norms (see also Tabibnia et al., 2008, for brain imaging results interpreted in this direction). Thus, ultimatum bargaining might encompass a conflict between the *short-run* goal of fairness and the *long-run* goal of self-interest. That is, while fairness could trigger immediate satisfaction, the maximization of one’s own payoff could to be related to long-run satisfaction^2^.

Yet other interpretations are possible. Espín et al. (2012) found that high DD is characteristic of free-riders who pay a cost to punish other free-riders in a one-shot public goods game. Free-riders’ punishment of other free-riders is considered to be motivated by competitive spite (i.e., spite emanating from positional concerns) because it increases the punisher’s relative standing and is hardly reconcilable with the moralistic or fairness-based motives which are assumed to be behind cooperators’ punishment of free-riders (Shinada et al., 2004; Falk et al., 2005; Eldakar et al., 2007). According to such a multi-dimensional interpretation of punishment behavior (Falk et al., 2005; Gächter and Herrmann, 2009), this finding suggests that the punishment decisions which respond to short-run psychological forces are not based on fairness but rather on competitive spite. However, those forces apparently have nothing to do with emotional reactions to aversive stimuli (since, prior to punishing, a free-rider has experienced neither an unfair treatment nor a disadvantageous relative position) but are instead linked to the personal satisfaction of outcompeting others, which can operate as a short-run incentive.

Since the rejection of a low offer in the UG both imposes the fairness norm and increases the relative standing of the responder, rejections could be driven by either fairness (normative) or spiteful (competitive) desires (Kirchsteiger, 1994; Carpenter, 2003; Falk et al., 2003). Thus, similarly to punishment behavior in the public goods game, looking exclusively at the rejection behavior of an individual is not enough to unequivocally infer her motives. Individuals’ strategies should be more broadly analyzed. In light of the findings of Crockett et al. (2010), where impatient subjects were more likely to reject low offers, and of Espín et al. (2012), where impatient punishers behaved uncooperatively themselves, a direct way to solve the puzzle is by looking into whether the “impatient responders” comply with the fairness norm when playing as proposers.

### Hypotheses

In this paper, we address this issue by analyzing how individuals’ DD relates to their behavior in both roles of the one-shot UG. If impatient subjects are found to reject low, unfair offers as responders and to propose high, fair offers as proposers, it can be argued that fairness goals respond to short-run incentives in the context of the UG. Hence:

**Hypothesis 1** states that *short-run orientation predicts fairness-based behavior* in ultimatum bargaining.

Indeed, fairness—either observed or imposed—is psychologically rewarding (de Quervain et al., 2004; Tabibnia et al., 2008; Crockett et al., 2013; see Tabibnia and Lieberman, 2007, for a review) and fair outcomes have been shown to activate various areas in the neural circuitry of reward (e.g., the striatum and the orbitofrontal cortex) which are typically also engaged in the computation of the subjective value of different alternatives when individuals make intertemporal decisions (e.g., Kable and Glimcher, 2007). According to Hypothesis 1, fair outcomes would be linked to non-monetary values (considered to function as motivational incentives; see Ruff and Fehr, 2014) that are perceived to be arising sooner, or to be less lasting, than those linked to other outcomes of the UG—such as, for instance, earning some money through the acceptance of a low offer or dominating the other player by means of offering her a low amount. So, deciding between different alternatives would require the evaluation of short- (fairness-related) and long-run satisfaction sources (possibly linked to self-interest). Such evaluation would lead impatient individuals to behave more fairly than patient individuals in both roles of the game.

On the other hand, it is known that the individuals’ payoffs relative to others rather than in absolute terms are associated with activation in reward areas of the brain (mainly striatal) when social interactions take place in competitive frameworks (Fliessbach et al., 2007; Dvash et al., 2010; Bault et al., 2011; Lindner et al., 2014), where there are positional or status concerns. Hence, for individuals interacting in a competitive environment, all things equal, satisfaction increases as the counterpart’s payoff decreases, meaning that reducing other’s payoffs should be psychologically rewarding. Bargaining processes might indeed create such an environment (Loewenstein et al., 1989). If these competition-based hedonic feelings (i.e., the satisfaction derived from outcompeting others) represent short-run incentives in the context of the UG, we would expect that in both roles of the game impatient subjects display spite-based behavior that reduces their partners’ payoffs, that is, they would reject low offers as responders and make low offers themselves as proposers. Hence:

**Hypothesis 2** states that *short-run orientation predicts spite-based behavior* in ultimatum bargaining.

In short, this hypothesis builds on Espín et al.’s (2012) findings and argues that the decision to play the UG harshly responds to the short-run hedonic value that individuals derive from outdoing others^3^. This behavior would make impatient individuals less likely to reach an agreement with their partners, thus risking the destruction of the pie for the sake of reducing the other party’s payoff.

This same behavior, however, cannot only be explained by spiteful preferences but also in terms of the ability of individuals to maximize their own payoffs, in particular, to strategically adapt their own decisions to the behavior of others. *Strategic behavior* may require cognitive and computational abilities. That is, it could be that impatience goes along with a diminished capacity to anticipate that offering a low amount (as a proposer) will potentially lead to a rejection and that setting a high punishment threshold (as a responder) means losing potential earnings. In fact, impatience has been related to low cognitive abilities (e.g., Burks et al., 2009; Dohmen et al., 2010). In such a case, impatient subjects would adopt less adaptive strategies, thus earning lower payoffs than the average due to their inability to anticipate others’ decisions^4^. Conversely, spiteful preferences are not focused on achieving a particular payoff for oneself but instead on reducing others’ payoffs. Thus, if spite is indeed what underlies the decisions of impatient subjects, we might observe that it is not the impatient subjects themselves but rather their interacting partners who end up with low payoffs. An analysis of the subjects’ earnings will allow us to explore this issue.

### Empirical Strategy

To test these hypotheses we analyze data from a citywide survey-experiment (see Exadaktylos et al., 2013, for a detailed description), which contains a dual-role UG and a measurement of participants’ intertemporal preferences. All participants [*N* = 713 final observations, 386 females, average age 36.7 ± 16.6 (SD)] were inhabitants of Granada, Spain. The sample consisted of participants aged 16 years and over and it was representative in terms of the geographical location of households within the city, as well as age and gender of the population. Participants made their experimental decisions anonymously from their own households in the presence of two monitors. Their (mathematical) cognitive abilities, risk preferences and extensive socio-economic information were also gaged [see Supporting Information (SI)].

The participants completed two complementary DD subtasks with six decisions each. The first subtask involved a 1-day delay, whereas the second implied a 6-months delay. The intertemporal preferences elicited over these delays will be referred to hereafter as short-run and long-run DD, respectively. This will serve us to check whether DD elicited over different time horizons may result in different associations with behavior (see Materials and Methods).

In the UG, participants made decisions as both proposers and responders in random order. We therefore obtained the strategy profile for each subject consisting of an offer as proposer and a minimum acceptable offer (MAO) as responder (see Materials and Methods). By using a UG task in which responders state their punishment threshold before learning the actual proposal, i.e., the strategy method (in contrast to the direct-response method, used by Crockett et al., 2010; see Brandts and Charness, 2011, for a review), we prevented role-specific time-dependent effects that would make the effect of DD on choices across roles incomparable. That is, had we used the direct-response method, participants would have learnt the final outcome from their own decisions as responders instantly, whereas as proposers they would have to wait for the responder’s decision in order to learn the outcome. This would have generated different delays between decision-making and outcome-realization across roles, which would have been problematic given that in our analysis we are jointly analyzing the effect of DD on both roles. Keeping instead by design identical delays between decision and outcome-realization across roles (by using the strategy method for the responder), our method preserves the main relationship of interest comparable.

## Results

### Delay Discounting and Behavior in the Ultimatum Game

**FIGURE 1:**
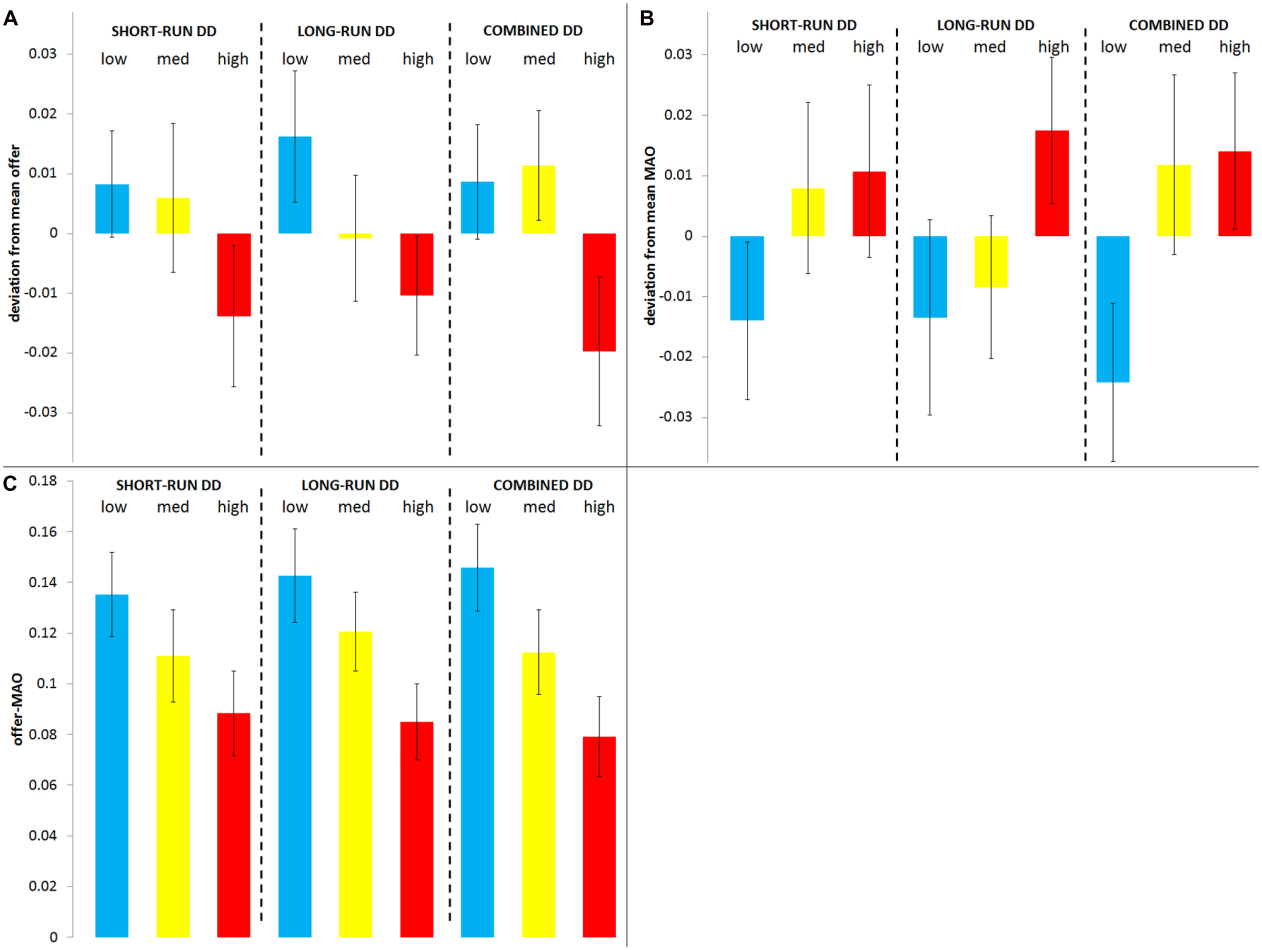
**Offer, minimum acceptable offer (MAO), and Offer-MAO by delay discounting (DD) groups**. Mean (±robust SEM clustered by interviewer) offer **(A)**, MAO **(B)**, and offer-MAO **(C)** by groups of DD. Both offers and MAOs are plotted in terms of their deviation from the mean behavior. From left to right and separated by dashed lines, the short-run, long-run, and combined DD appear split in terciles (“low,” “med,” and “high” for the first, second, and third tercile, respectively).

In **Figure 1** we show the mean (± robust SEM clustered by interviewer) offer (panel A), MAO (panel B), and offer-MAO (panel C) as a function of different DD characterizations. Short- and long-run DD are measured based on the number of impatient responses (from 0 to 6) the individual made for each of the two subtasks, while “combined DD” refers to the average of the above DD measures ([DDs+DDl]/2). For visual clarity we categorized individuals in three groups according to their DD (as in Espín et al., 2012) and plot offers and MAOs in terms of their deviation from the mean offer (0.462 ± 0.007) and MAO (0.350 ± 0.009), defined as a fraction of the pie. Positive deviations indicate above-average offers or MAOs in each case. From left to right and separated by dashed lines, the short-run, long-run, and combined DDs appear split in terciles (“low,” “med,” and “high” for the bottom, middle, and top tercile, respectively).

It can be observed that mean offers and MAOs vary along with DD groups. In particular, more impatient individuals appear to have on average lower offers and higher MAOs. Interestingly, these relationships are qualitatively similar regardless of the DD measure used. The effect of DD is even clearer on the difference “offer-MAO,” a variable that serves as a measure of the margin of agreement each individual allows: the larger one’s offer and the smaller one’s MAO (thus, the larger the difference), the more likely the individual is to agree with others thus preventing the destruction of the pie. We observe that more impatient individuals allow a lower margin of agreement.

To test the statistical significance of these relationships, we performed a series of regressions where the raw DD scores are introduced as continuous explanatory variables (mean values of the three UG variables across raw DD scores are displayed in Supplementary Figures S3 and S4). **Table 1** presents the estimates of the impact of DD on UG behavioral strategies in columns (1)–(3). Each cell contains estimates from one separate regression, with the variable at the top of the column as the dependent variable. In all regressions we control for socio-demographic variables (age, gender, marital status, household income, and educational level), cognitive abilities, risk preferences, and order effects in decisions as possible confounding factors. Robust standard errors are clustered on interviewers in order to account for the non-independence of the observations gathered by the same pair of interviewers. OLS estimates are shown for comparability of coefficients (other regression methods like Tobit or ordered models yield similar main results and are available upon request from the authors) and two-tailed *P*-values are presented in brackets. Asterisks denote significant estimates. The complete regressions can be found in Supplementary Tables S1–S4.

**Table 1:** Impact of DD over UG strategies and expected payoffs.

Dependent vars.	Offer	MAO	Offer-MAO	*Own payoff*	*Other’s payoff*
	(1)	(2)	(3)	(4)	(5)
Short-run DD	-0.0294	0.0428^∗^	-0.0722^∗∗^	-0.0115	-0.0252^∗^


	(0.157)	(0.052)	(0.024)	(0.131)	(0.053)
Long-run DD	-0.0324^∗∗^	0.0393^∗^	-0.0717^∗∗∗^	0.0018	-0.0269^∗∗∗^


	(0.041)	(0.051)	(0.004)	(0.785)	(0.009)
Combined DD	-0.0437^∗∗^	0.0581^∗∗^	-0.1018^∗∗∗^	-0.0070	-0.0369^∗∗∗^


	(0.038)	(0.017)	(0.002)	(0.404)	(0.006)
Highdd vs. lowDD	-0.0281^∗^	0.0431^∗∗∗^	-0.0711^∗∗∗^	-0.0069	-0.0231^∗∗^


	(0.052)	(0.005)	(0.001)	(0.215)	(0.013)

*The estimated coefficients for different DD characterizations as explanatory variables are shown in rows. Dependent variables are expressed as a fraction of the pie (€20). Each estimate refers to a different OLS regression with robust standard errors clustered by interviewer (108 groups) and controlling for age, gender, married, household income, educational level, mathematical cognitive abilities, risk preferences, and order effects. N = 713, except for the last row where n = 488. oxP-values are shown in brackets (two-tailed). ^∗^P < 0.10, ^∗∗^P < 0.05, ^∗∗∗^P < 0.01. The complete regressions are presented in Supplementary Tables S1–S4*.

Different characterizations of DD are presented in rows. The first, second and third rows show the effect of short-run, long-run, and combined DD, respectively, on the dependent variables. To facilitate interpretation, the three measurements of DD are normalized to the interval [0, 1] so that the reported coefficients refer to the difference between the least and the most impatient individuals, according to each measure. Finally, “highDD vs. lowDD,” in the fourth row, is a binary variable taking the value 1 if the individual belongs to the top 33% and 0 if the individual belongs to the bottom 33% of the distribution of “combined DD.” Observations falling in the central 33% are missing for the analyses shown in the last row, hence the sample for this last exercise is reduced to *n* = 488.

Columns (1), (2), and (3) refer to regressions with the individual’s offer, MAO, and their difference as dependent variables, respectively. These variables are expressed as a fraction of the pie.

The impact of both short-run and long-run DD on offers [column (1)] is negative and quantitatively similar, but only reaches significance in the case of long-run DD. As we discuss in the SI, we cannot disentangle whether this difference is due to the fact that the presence of immediate payoffs in the short-run task reduces the predictive power of short-run DD [which would be in favor of the dual-valuation account of DD and at the same time prevent an explanation in terms of behavioral control (Figner et al., 2010)] or to the poor distribution of subjects’ responses in that subtask (Supplementary Figure S1). Note that, in any case, we can infer that neither present bias nor behavioral control is the *only* factor related to proposers’ behavior since these should not influence choices in the long-run DD subtask. A seemingly substitutive effect between short-run DD and long-run DD (when including both variables in a single regression their coefficients are still negative but lose significance; not reported) suggests that we will possibly obtain a better picture by combining both measures. In fact, the variable “combined DD” reports a slightly stronger effect on offers. The “highDD vs. lowDD” variable yields a similar result. Thus, the effect of DD on offers is negative, though rather small (between 2.81 and 4.37% of the pie, i.e., between 18.96 and 29.49% of one SD of the dependent variable, depending on the DD specification).

On the other hand, all the estimates of DD are positive and significant when the dependent variable is the individual’s MAO [column (2)]. Hence, we replicate the finding by Crockett et al. (2010) to the extent that more impatient responders are more likely to reject low offers. This result suggests that the inability to inhibit an immediate response to an aversive stimulus (i.e., a negative emotion; see Sanfey et al., 2003; Van’t Wout et al., 2006; Tabibnia et al., 2008) is not the only source of impatient responders’ rejections. The evaluation of short- and long-run goals should play a crucial role as well since (i) the relationship also arises under the “cold” strategy method and (ii) both the long-run DD—which should not depend on executive control—and the short-run DD similarly predict rejections. The effect of DD on MAOs is larger than its effect on offers but still quite small (between 3.93 and 5.81% of the pie, i.e., between 22.22 and 32.84% of one SD of the dependent variable).

Column (3) shows that the above relationships translate into a relatively strong effect of DD on “offer-MAO.” This means that the margin for agreement shrinks as DD increases. It will therefore be easier to shake hands with a patient individual. Specifically, these effects lie between 7.11 and 10.18% of the pie, that is, between 30.12 and 43.12% of one SD of the dependent variable. As in the case of offer and MAO, here, we observe as well that short-run and long-run DD are associated with the same patterns, and combining the two measures improves the model’s power of fit.

While **Figure 1** provides an illustrative picture with regards to the effect DD has on bargaining behavior, the estimated effects and statistical significance are properly obtained through regressions (**Table 1**). In particular, from both **Table 1**; **Figure 1** we obtain the following:

**Result 1**: short-run oriented subjects offer less in the UG;

**Result 2**: short-run oriented subjects reject more, that is, their MAO is higher.

Consistent with Results 1 and 2, short-run oriented subjects do not facilitate agreements. Specifically, the margin for agreement (offer-MAO) allowed by the most patient individuals virtually doubles that allowed by the most impatient individuals. (Note here that both patient and impatient groups display mean offers well above mean MAOs.)

Taken together, these results lead to a r*ejection of Hypothesis 1*, according to which impatience should predict individuals’ concern for fairness in either role of the UG, since the offers made by high-DD proposers are, on average, more unfair. In terms of the Fehr and Schmidt’s (1999) inequality aversion model, impatient individuals appear to exhibit stronger aversion to disadvantageous inequality and weaker aversion to (possibly even “negative aversion” to, i.e., preference for) advantageous inequality compared to patient individuals (proposers’ offers are not only driven by “pure” preferences but also by the avoidance of rejection, so it would be hard to conclude about the sign of advantageous-inequality aversion). In the context of the UG, this would imply that impatient individuals are keen on equalizing payoffs when they are below but unwilling to do so when they are above, which is consistent with spiteful preferences. Therefore we do find *support for Hypothesis 2*, according to which impatience should predict individuals’ spite-based decisions in the UG.

### Does the Final Outcome Relate to Individuals’ Delay Discounting?

As stated in the Introduction, however, the above results might also be explained if high DD predicts less strategic rather than more spiteful behavior. To disentangle the two, we now focus on the participants’ payoffs. We simulated a perfect random matching between participants (i.e., like a round-robin tournament where everybody plays once against everybody in each role) resulting in 1,424 (712 interacting partners ^∗^ 2 roles) simulated interactions per subject, and computed their expected (mean) payoffs as a proxy for reproductive fitness. From an evolutionary viewpoint, this method will actually give us an appropriate measure for the adaptiveness of the strategies adopted in the UG, since the probability of matching with each of the other participants across the city was identical. We obtained the expected payoff per interaction of each individual (“*own payoff*”) and that of the other individuals when interacting with her (“*other’s payoff*”), which were calculated from the actual distribution of individual strategies in the sample (see Materials and Methods). Recall that these expected payoffs are calculated for each individual based on her decisions in both roles (i.e., the average across the two roles). Under the interpretation that impatience goes along with an inability to figure out how the distribution of others’ strategies in the sample looks like or to appropriately respond to others’ behavior (i.e., impatience reflects impaired strategic adaptation), and assuming that patient and impatient individuals are equally self-interested, impatient individuals should, by definition, earn less money. If, on the other hand, patient and impatient individuals are equally good at anticipating others’ strategies but impatience predicts a higher willingness to reduce others’ payoffs (i.e., Hypothesis 2), we should expect that it is the counterpart’s payoff, instead of the own payoff, that negatively correlates with the impatience of the decision-maker.

**FIGURE 2:**
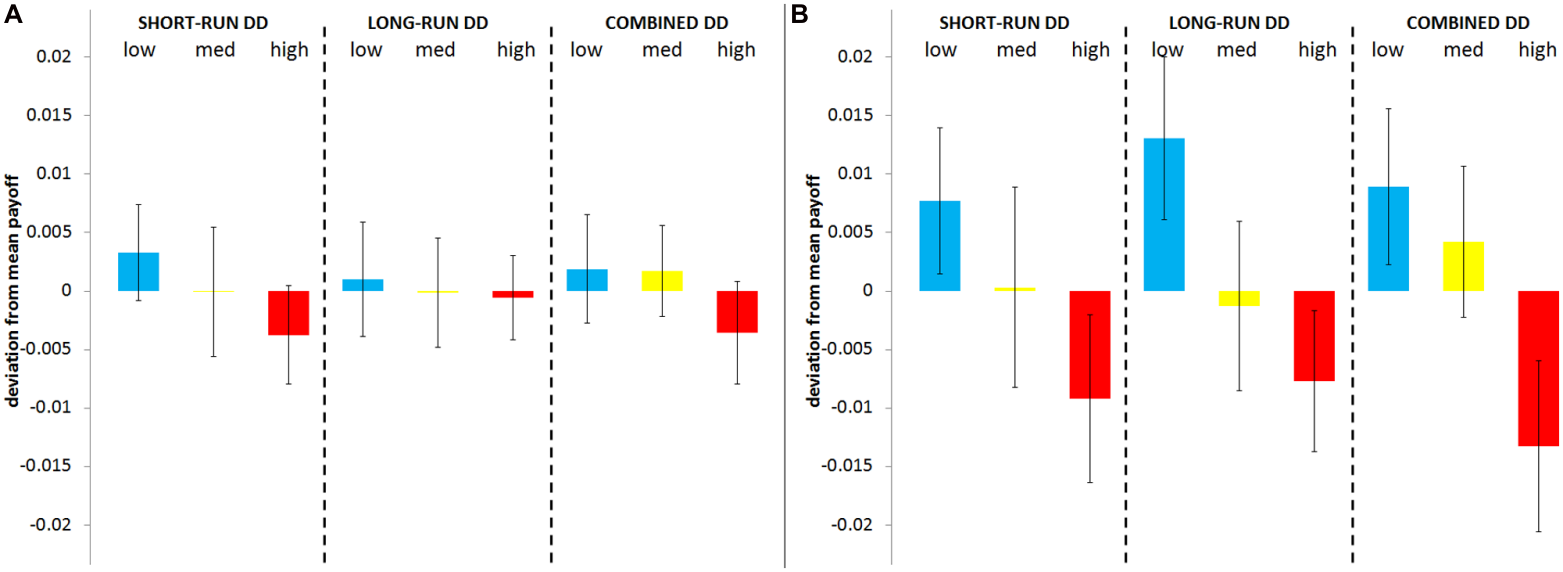
**Own and other’s payoff by DD groups**. Mean (±robust SEM clustered by interviewer) *own payoff* **(A)** and *other’s payoff* **(B)** by groups of DD (same groups as in **Figure 1**). Both own and other’s mean payoffs are plotted in terms of their deviation from the mean payoff.

In **Figure 2**, *own payoff* (panel A) and *other’s payoff* (panel B) are expressed as a fraction of the pie and plotted in terms of their deviation from the mean payoff (0.430 ± 0.003). The same categorizations of DD of **Figure 1** are employed.

It is nicely illustrated that the struggle will be fiercer with impatient interacting partners since, as impatience increases, there is a rather strong decrease in *other’s payoff*, whereas the variation in *own payoff* appears to be less pronounced. Again, the statistical significance of these relationships is assessed through regression analyses with DD as a continuous explanatory variable (mean values of *own payoff* and *other’s payoff* across raw DD scores are displayed in Supplementary Figure S5).

In column (4) of **Table 1** we display the estimates for regressions with the expected *own payoff*, expressed as fraction of the pie, as the dependent variable. None of the DD specifications results in significant estimates. Thus, based on the analysis of the expected payoffs we do not find support for the argument that high discounters were simply unable to apply an advantageous (i.e., more adaptive) strategy. Participants with high DD did not earn less (or more) than participants with low DD, as participants’ payoffs were not significantly related to their DD.

Interestingly, however, a salient result is that DD impacts negatively and significantly on *other’s payoff* according to all DD specifications [column (5)]. That is, the higher the DD of an individual’s interacting partner, the less she is expected to earn from that interaction. The total effect of DD on *other’s payoff* ranges between 2.31 and 3.69% of the pie. Putting these findings into an evolutionary perspective and considering the expected payoffs as a measure of potential reproductive fitness, this effect, although seemingly small, is in fact profound. The 99th and 50th percentiles of the distribution of *own payoff* are 0.482 and 0.451, respectively; hence, a mere 3.1% reduction in the payoff is sufficient to depress an individual’s fitness from the top to the median part of the distribution.

Therefore, the strategies adopted by patient and impatient individuals are equally adaptive but the effects these strategies have on others are substantially different: individuals (either patient or impatient) will get lower payoffs from their interactions with impatient individuals than from their interactions with patient individuals. It follows that individuals facing impatient bargaining partners more often than others will have lower expected payoffs and hence lower survival probabilities as well.

Based on **Figure 2** and the regression analyses of **Table 1** we therefore conclude:

**Result 3**: short-run oriented subjects do not earn less money;

**Result 4**: the partners of short-run oriented subjects earn less money.

Given these results, it appears that high-DD individuals are not less strategic but indeed more spiteful. In other words, the alternative rationale (i.e., that high-DD individuals’ decisions reflect a diminished capacity to maximize their own payoffs in a strategic setting) fails the test of the individuals’ payoffs. Therefore, from Results 1 to 4, only the spite account for the observed behavior of impatient individuals (as predicted by Hypothesis 2) finds support.

Finally, we look into the role of cognitive abilities in order to shed more light on how behavior and payoffs are linked with each other. Although cognitive ability, as measured by the number of correct answers to five mathematical questions, and impatience are unrelated in our sample (regressions using a variety of model specifications, both with and without control variables, as well as parametric and non-parametric correlation analyses all produce *Ps* > 0.3; not reported), previous work suggests that there might be a potential confound (Burks et al., 2009; Dohmen et al., 2010). This lack of relationship may have to do with methodological differences across studies (e.g., the specific cognitive measures employed). Supplementary Tables S1–S4 in SI reveal that subjects endowed with higher cognitive skills achieve a higher *own payoff* [*P* = 0.009 in the regression using “combined DD”; Supplementary Table S3, column (4)]. However, higher cognitive abilities also predict higher MAOs [*P* = 0.029; column (2)], which is at odds with the payoff maximizing strategy, i.e., setting MAO either to zero or to the smallest possible amount. In addition, cognitive abilities do not predict offers being closer to the equal split [*P* > 0.7; column (1)], which in our sample would be the payoff maximizing strategy. That is, subjects with higher cognitive abilities succeed in achieving larger total payoffs, as one would expect, but at the same time they do not manifest payoff maximizing behavior when analyzing each role separately. Such an observation is important insofar as it shows that the relationship between the behavioral predictors (cognitive abilities and DD) and the final payoffs is not so clear-cut. Concretely, in our case, what determines subjects’ payoffs is the whole behavioral profile coming from both choices in the UG and the actual distribution of choices across the city. Thus, observing higher DD to predict both lower offers (Result 1) and higher MAOs (Result 2) but not lower payoffs (Result 3) should not be considered a puzzle.

## Discussion

The aim of the study was to explore the relationship between individuals’ intertemporal preferences and bargaining behavior in the UG. Low offers clearly violate fairness norms but, at the same time, they provide the perfect ground for spiteful preferences to manifest (Kirchsteiger, 1994; Jensen, 2010). Our design, in which subjects play both roles of the UG, allows us to uncover the preferences that underlie the link between impatience and rejection behavior (Crockett et al., 2010). We find that high DD predicts spiteful rather than fair strategies in both roles. These spiteful strategies involve the rejection of disadvantageous, “unfair” splits, but also the proposal of advantageous, “unfair” splits. Quite importantly, this behavioral pattern by high discounters does not lead them to earn less (i.e., their strategies are not less adaptive), but they result instead in significantly lower payoffs for those who interact with them. This is consistent with the observation of Marlowe et al. (2011) that hunter-gatherers of smaller societies, assumed to strongly discount the future (Woodburn, 1980), show more spiteful behavior in the UG. Thus, while reducing others’ payoffs appears to work as a short-run proximate motivation in ultimatum bargaining, future research should try to determine which outcome(s) might represent the long-run goal(s). According to our findings, the unwillingness to harm others, or the desire to increase others’ payoffs or the joint surplus (reaching an agreement implies that the pie is not destroyed) are the most obvious candidates. Short-run orientation has indeed been related to both aggressive (Nelson and Trainor, 2007; Espín et al., 2012) and uncooperative (Curry et al., 2008) patterns.

We interpret this result as evidence that spite-based behavior responds to short-run psychological satisfaction. However, it may be argued that living environments favoring local (vs. global) competition for resources could impose social-ecological pressures for the selection of both spiteful and short-run oriented preferences separately (Gardner and West, 2004; Daly and Wilson, 2005; Hill et al., 2008). Thus, both traits might serve as cognitive adaptations to environmental cues of local competition signaling a strong link between individuals’ reproductive success and their short-run relative standing. In fact, it has been found that exposure to harsh social conditions during childhood predicts later seemingly spiteful behavior in a repeated prisoner’s dilemma game (McCullough et al., 2013), supporting the notion that social preferences are importantly shaped by daily life circumstances (Van Lange et al., 1997; Rand and Kraft-Todd, 2014). Similarly, unpredictable developmental environments may lead to short-run orientation and other “impulsive” behaviors (Hill et al., 2008). This line of argument could raise concerns on whether the way we interpret the relationship between impatience and spiteful behavior is the only possible one (even though both views are not necessarily in contradiction), or if instead there exists such an unobserved third variable driving the result. For example, other personality traits, which we have failed to measure, might be also affecting bargaining behavior. Although we control for a large set of socio-economic characteristics of subjects in the statistical analyses, this possibility cannot be completely ruled out in a correlation study. A more systematic approach to such potentially underlying processes would thus be an interesting endeavor for future research (Peysakhovich and Rand, in press, for instance, provide a clever experimental manipulation that could be adapted to systematically study these processes).

Based upon previous research (Espín et al., 2012), our findings suggest that rejection behavior in the UG might better resemble the spiteful punishment by free-riders in terms of its psychological foundations than the moralistic or fairness-based punishment by cooperators. In effect, the bargaining—intrinsically conflictive—nature of the UG could be generating a competitive environment, where outperforming the other player is a primary goal (Fliessbach et al., 2007; Dvash et al., 2010; Bault et al., 2011). In ultimatum bargaining, both players can make use of their own forces to prevent the other player from achieving her goals, thus offering a natural context for the expression of dominance behavior (Jensen, 2010), which is deeply rooted in early human cognitive development (Thomsen et al., 2011; Mascaro and Csibra, 2012). Such an interpretation is to some extent coherent with recent findings indicating that the rejection of low offers may reflect a tendency to avoid being subjugated to the other player (Yamagishi et al., 2012).

Along these lines, Crockett et al. (2013) found that serotonin-depleted participants were more likely to reject low offers in the UG, but tended to be less likely to punish low offers as third-party observers (which is considered to reflect a clear concern for social fairness, see Fehr and Fischbacher, 2004). Moreover, reducing subjects’ serotonin levels was found to increase the psychological satisfaction (as measured by striatal activation) of rejecting low offers in the UG, but to decrease the satisfaction associated with receiving fair offers. These results indicate that reduced serotonergic activity predicts retaliatory motives behind the rejection of low offers in the UG rather than an enhanced preference for fairness. Our findings are consistent with those of Crockett et al. (2013) insofar as there is an intimate link between low serotonin levels and high DD (Schweighofer et al., 2008; Crockett et al., 2010). Thus, responders’ retaliatory behavior triggered by serotonin depletion could reflect a short-run oriented, spiteful desire to reduce the proposer’s payoffs.

Taken together, these studies indicate that in the UG not all of rejection decisions are driven by the notion of fairness. Accordingly, they add to a growing literature on social dilemma games (Shinada et al., 2004; Falk et al., 2005; Herrmann et al., 2008; Gächter and Herrmann, 2009; Rand and Nowak, 2011; Espín et al., 2012), giving support for the presence, not only of fairness concerns, but also of competitive spite—a pro-self rather than pro-social sentiment—as a key psychological ingredient behind costly punishment.

In social dilemmas, the punishment of free-riders by cooperators is considered a second-order cooperative behavior as it is beneficial for the group (Fehr and Gächter, 2002; Gächter et al., 2008) though not for the punisher (Dreber et al., 2008) in the long term. However, both empirical (Herrmann et al., 2008) and theoretical (Rand et al., 2010; Rand and Nowak, 2011) evidence suggests that, under specific circumstances, spiteful punishment by free-riders may dramatically challenge the dynamics of cooperation and the long-run social efficiency (for an overview, see Sylwester et al., 2013). Therefore, special care has to be taken when using the standard UG (where one cannot know whether or not the punisher wants to enforce fairness or take a spiteful, competitive attitude) as a device to study peer punishment and, on top of that, when building theories on how individuals, institutions or groups enforce the relevant social norms based on results from rejection behavior in this game. Future research should take into account that hyper-competitiveness (Jensen, 2010) might be as fundamental to the complexity of human social behavior as ultra-sociality (Richerson and Boyd, 1998).

### Limitations

Some potential limitations of the present study should be acknowledged. First, as already mentioned, the correlation nature of our analysis does not allow drawing firm conclusions regarding the causality of the observed relationships, leaving open alternative interpretations.

Secondly, the hypothetical nature of reward in the discounting elicitation task should be considered. In particular, even though it does not seem to be the case in intertemporal choice (e.g., Johnson and Bickel, 2002), hypothetical (vs. real) payments may admittedly affect behavior. Indeed there are many well-studied examples where this is the case (Slovic, 1969; Holt and Laury, 2002; Harrison and Rutström, 2008; El Harbi et al., 2015; see, however, the review by Camerer and Hogarth, 1999, the main point of which is that “it depends”). On the other hand, hypothetical reward might potentially be better suited in a face-to-face experiment like ours that involves choices with immediate as well as delayed payments, since keeping both the transaction costs and the level of payment-uncertainty (i.e., whether the payment will actually be made) equivalent across the different choices is a particularly difficult task. In these cases, there are good reasons to expect that hypothetical reward might even lead to less biased estimations (see Read, 2005, for an interesting discussion on the appropriateness of real incentives). However, it would be important for future research to test the extent to which the nature of incentives matters for the relationships studied in this paper.

Thirdly, it should be noted that due to the restrictions imposed by the features of our survey-experiment, the DD task employed was not designed to allow for a precise estimation of the individuals’ discount rates. Whenever possible researchers should try to elicit intertemporal preferences more precisely (see the methods used, for instance, in Burks et al., 2009; Andreoni and Sprenger, 2012; Andersen et al., 2014; Bradford et al., 2014).

## Materials and Methods

### Procedures

All participants were respondents of a wider survey-experiment that was conducted from November 23rd to December 15th 2010 in the city of Granada, Spain, using a door-to-door approach. The first part of the survey included a questionnaire, lasting about 20 min, gathering socio-economic information, as well as risk preferences and cognitive abilities. Participants then completed the hypothetical delay-discounting task and upon completion they made their monetarily incentivized experimental decisions. Matching and payments took place within the next 2 weeks. Interviewers were last-year university students, who worked in pairs. Detailed information regarding the protocol and the exact content of the survey-experiment can be found in the SI (see also Exadaktylos et al., 2013).

### The Delay-Discounting Task

In the discounting task, participants had to state their willingness to wait in order to receive a hypothetical monetary payoff. The participants responded verbally to the decisions presented (also verbally) by one of the interviewers and their responses were written down by the second interviewer. In contrast to the UG, decisions in this task were not incentivized with money for technical and logistical reasons associated to field investigation (delayed payments, re-contact participants on a specific date, etc.). Most importantly, previous studies have shown that real (versus hypothetical) incentives do not change the distribution of individual responses in DD tasks either within or between subjects (Johnson and Bickel, 2002; Madden et al., 2004; Lagorio and Madden, 2005; however, see Coller and Williams, 1999) and that intertemporal choices over both real and hypothetical reward result in identical brain activations (Bickel et al., 2009). However, since in contrast to the lab, the field cannot always guarantee maximum control, subjects paying less attention to a purely hypothetical task (Frederick et al., 2002) cannot be ruled out and can potentially generate nosier data. On the other hand, we expect that this issue was eased off by the use of a large sample size. Thus, while we do not think that the use of hypothetical incentives has affected the sign of the relationships under study, it might have affected their size. In fact, as reported in Section “Results,” some of our observed effects are not large in size, and hypothetical incentives may be partly responsible.

Participants were asked to choose between sooner-smaller reward and larger, but more delayed reward in a series of binary decisions. The larger the delayed amount needed for “convincing” an individual to wait, the higher her DD score (i.e., her impatience). We used the following protocol:

• The short-run DD was measured by having participants choose between €5 available “today” and €5+X (X = €0, …, €5) to be received “tomorrow.”
• For the long-run DD, the six choices were between €150 delayed by 1 month and €150+X (X = €0, …, €100) delayed by 7 months (see SI).

The average number of impatient responses (i.e., the number of times individuals chose the sooner-smaller reward, out of six) was 2.75 ± 0.127 (robust SEM clustered by interviewer to account for dependency between the observations gathered by the same interviewers, leaving a total of 108 independent groups) in the short-run subtask and 3.16 ± 0.087 in the long-run one (see Supplementary Figure S1 for the distribution of choices in the DD subtasks).

According to dual-valuation neurobiological theories of DD (McClure et al., 2004, 2007; built on the models introduced by Phelps and Pollak, 1968; Laibson, 1997), these two measures could capture different components of the psychological evaluation of delayed reward because the long-run subtask did not involve trading-off immediate payoffs. That is, the so-called present bias should affect subjects’ choices in the short-run but not in the long-run subtask (see SI)^5^. In contrast, single-valuation theories (e.g., Kable and Glimcher, 2007) claim for a unique process underlying the evaluation of delayed and immediate reward. While they differ in the number of interacting processes at play, both of these accounts locate the underlying neural system of intertemporal choice within the *valuation* regions of the brain (such as the ventral striatum and the ventromedial prefrontal cortex).

A different strand of research, however, highlights the distinction between “impulsive action” and “impulsive choice” (see Broos et al., 2012; Paterson et al., 2012; Bari and Robbins, 2013 for a review)^6^. Accordingly, impatient choices in DD tasks can result not only from a higher subjective valuation of the sooner over the latter option but also from an inability to overcome the temptation of choosing the former, that is, to exert *behavioral control* over a prepotent response, irrespective of its relative valuation (Figner et al., 2010; Hare et al., 2014; Steinbeis et al., 2014). Behavioral control has been traditionally related to *executive* brain areas in the lateral and particularly the dorsolateral prefrontal cortex (Miller and Cohen, 2001). Our task should also allow us to shed light on the potential role of behavioral control since its effect on intertemporal choice appears to be restricted to trials were the sooner option is immediate (Figner et al., 2010). Thus, participants’ choices in the long-run subtask should not be influenced by executive but only by valuation processes.

In sum, if we find that both short-run and long-run DD relate similarly to behavior in the UG, it would be difficult to argue that the effect of DD is due to individual differences in either present bias or the capacity to exert behavioral control. The Spearman’s rank order correlation between the number of impatient responses in short-run and long-run subtasks is 0.302 (*P* < 0.001). This far-from-perfect correlation opens a door for the two measures to be actually capturing different psychological constructs and, consequently, having distinct associated behaviors (see SI for a discussion on this issue). That said, it should be noted that the two subtasks also involve different payoff magnitudes (€5– €10 vs. €150– €250) and time delays (1 day vs. 6 months). Therefore, a potential magnitude effect (i.e., larger payoffs are discounted less than smaller payoffs, see for example Green and Myerson, 2004) or differences in time perception (i.e., time is perceived non-linearly, see Bradford et al., 2014) could also lead to differences across subtasks that are not strictly due to dual-valuation or differences in behavioral control^7^.

In the literature on DD, there is a long-lasting discussion regarding which particular functional form best characterizes individuals’ discounting. While some scholars suggest that discount rates are constant (i.e., exponential, time-consistent discounting) others argue that discount rates decline over time [i.e., a (quasi-) hyperbolic functional form, which involves time-inconsistent preferences; see Frederick et al., 2002; Green and Myerson, 2004]. Due to the restrictions imposed by the nature of our survey-experiment, however, the discounting elicitation task was not designed to allow us estimate the parameters of the individuals’ discount functions. Consequently, an in-depth analysis of the *prevalence* of constant versus declining discount rates cannot be performed.

### The Ultimatum Game

In the UG, all participants made decisions as both proposers and responders in random order. The pie to split was €20 (≈$27). A double blind procedure was employed by means of a decision card that was introduced in a sealed envelope by the participant after writing down her decisions. As proposers, participants were asked to state which share of the €20 (in 10% increments) they wanted to offer to an anonymous partner. As responders, participants were asked to accept or reject each of the following proposals (proposer’s payoff, responder’s payoff): (€20, €0), (€18, €2), (€16, €4), (€14, €6), (€12, €8), (€10, €10), that is, the strategy method was employed (Mitzkewitz and Nagel, 1993). This allowed the elicitation of each participant’s MAO.

Apart from the obvious advantage of eliciting the full strategy profile for every participant, employing the strategy instead of the direct-response method allowed for the following crucial features of our design. First, as already discussed in the Introduction, the direct-response method would have eventually generated different time-dependent effects across roles, thus preventing a clear interpretation of the results. Second, under the strategy method, the outcome of the decision a participant made first (either proposer or responder, randomly chosen) could not have influenced her behavior at the second decision. Third, an alternative rationale behind a potential positive relationship between rejections and impatience (predicted by both Hypothesis 1 and 2) is based on the inability of impatient responders’ to inhibit prepotent responses^8^. The strategy method arguably reduces the scope for such an interpretation since responders decide in a rather “cold” state. In any case, our DD task may allow us to disentangle if the effect of DD on rejections is driven by behavioral control (in which case rejections should not be predicted by the long-run DD).

Hence, through our experimental protocol we obtained the strategy profile for each subject consisting of an offer as proposer and a MAO as responder. After making their decisions, participants were randomly paired in order to calculate the real payoffs according to their chosen strategies and those of their counterparts. Thus, subjects were playing a one-shot, dual-role, simultaneous UG (see Supplementary Figure S2 for the distribution of choices in the game). One out of every ten participants was randomly selected for real payment (see SI).

### Expected Payoffs

In order to explore the relationship between an individual’s DD and both her own and her partners’ earnings, we will analyze the participants’ expected payoffs. This analysis will help to uncover potential differences in strategic (vs. spite-based) behavior across DD groups. As described by Iranzo et al. (2012) and Rand et al. (2013), the average payoff for individual *i* (with offer = *o_i_* and MAO = *m_i_*) after interacting in both roles with individual *j* (*o_j_*, *m_j_*) is given by (a) ½(1-*o_i_*+*o_j_*) if *o_i_* ≥ *m_j_* and *o_j_* ≥*m_i_*; (b) ½(1-*o_i_*) if *o_i_* ≥*m_j_* and *o_j_* < *m_i_*; (c) ½*o_j_* if *o_i_* < *m_j_* and *o_j_* ≥*m_i_*; and (d) 0 if *o_i_* < *m_j_* and *o_j_* < *m_i_*. Hence, the expected “*own payoff*” for individual *i* is calculated by weighting each of these four possible payoffs by the probability of that specific case occurring within our sample (i.e., its relative frequency). Analogously, the average payoff for individual *i*’s partner (i.e., individual *j*; the “other”) in the same cases is (a) ½(1-*o_j_*+*o_i_*); (b) ½*o_i_*; (c) ½(1-*o_j_*); and (d) 0. The expected “*other’s payoff*” for individual *i* is calculated by assigning weights (same as above) to the latter payoffs, and reflects the payoff an individual expects to get when interacting with individual *i* (see SI, where we also provide a numerical example of how the expected payoffs are practically calculated).

### Ethics Statement

All participants in the experiments reported in the manuscript were informed about the content of the experiment prior to participating. Identical instructions were read aloud by the interviewers. Since literacy was not a requirement to participate (this was necessary to obtain a representative sample because such a requirement would have excluded by design a share of a population) we could not ask participants to read and sign the IC. Oral informed consent was obtained from all participants included in this paper. Those who did not accept to sign did not continue the experiment. Anonymity was always preserved (in agreement with Spanish Law 15/1999 on Personal Data Protection) by randomly assigning a numerical code to identify the participants in the system. No association was ever made between their real names/addresses and the results. As is standard in socio-economic experiments, no ethic concerns are involved other than preserving the anonymity of participants. 9Note that actual payoffs do not provide the most accurate mapping between payoffs and strategies. The recombination of individuals’ strategies in simulated interactions indeed allows for better estimations. A similar approach was applied for instance in Mitzkewitz and Nagel (1993) and Chen and Li (2009). See also Mullin and Reiley (2006) for the application of “recombinant estimation” in other settings.

This procedure was checked and approved by the Vice-dean of Research of the School of Economics of the University of Granada; the institution hosting the experiments. At that time not any official IRB committee was established at the School of Economics.

## Author Contributions

All authors contributed equally to all parts of the research.

## Conflict of Interest Statement

The authors declare that the research was conducted in the absence of any commercial or financial relationships that could be construed as a potential conflict of interest.

## Footnotes

1 Technically speaking, in order for an agent to be defined as spiteful, it is required that the first derivative of his/her utility with respect to others’ payoffs is *always* negative, irrespective of both their behavior and the payoff distribution: the “more negative” the impact of others’ payoffs on utility the more spiteful the individual. Therefore, we refer to spite (also referred to as spitefulness; see Fehr and Schmidt, 2006) as a proximate motivation to reduce others’ payoffs in order to increase one’s own utility. The maximization of the individual’s relative payoff (own payoff *minus* other’s payoff), emanating from competitive preferences or a competitive value orientation (Messick and McClintock, 1968; Van Lange et al., 1997; Charness and Rabin, 2002), could thus be considered as a particular form of spite. Note, however, that in some research areas, spite is often used to refer to those behaviors that merely impose a cost on others, regardless of whether reducing others’ payoffs is the actual motivation (that is, the functional approach). See Jensen (2010) for a discussion on the different definitions of spite.

2 As Crockett et al. (2010, p. 870) point out, “[…] the emotional satisfaction derived from punishing (e.g., rejecting an unfair offer) is immediate, while the monetary benefit of not punishing (e.g., accepting an unfair offer) is delayed until the end of the experiment.”

3 In the Section “Discussion,” we provide an alternative (but not exclusive) rationale that does not strictly require the desire to reduce others’ payoffs to be associated with short-run satisfaction. Instead, it is argued there that adaptation to particular living conditions might result in the selection of present-oriented and spiteful preferences separately.

4 Note that the behavior predicted by Hypothesis 1 for impatient proposers (i.e., making a fair offer) can be accounted for by strategic self-interest as well, but the behavior predicted for impatient responders (i.e., setting a high punishment threshold) cannot. So, only the predictions of Hypothesis 2 are congruent with the existence of differences between patient and impatient individuals in terms of strategic adaptation.

5 Interestingly the anterior cingulate cortex, a brain region frequently associated with the presence of response conflict (e.g., Barch et al., 2001), has been found to be more strongly activated during choices between immediate and delayed reward than between only delayed reward (McClure et al., 2007). This might reflect the conflict between the two valuation systems, which should interact only when one of the options is immediate.

6 “Impulsive choice” refers to choosing the sooner option in DD tasks, while “impulsive action” refers to the inability to inhibit a prepotent response.

7 Reviewer #3 called our attention to these two potential confounding factors and suggested that therefore the employed task does not permit making any inferences regarding dual-valuation theories whatsoever. While acknowledging the limitations of the task, we do consider that we can discuss about the dual-valuation theories since their main argument is that making intertemporal choices where one of the options is immediate engages—by definition—both valuation systems, while choosing between two delayed options engages only one of the two systems (see SI). Yet, due to these limitations we have avoided making any strong claims in this regard when discussing the results.

8 It must be noted, however, that neurobiological results on the influence of executive function on UG rejections under the direct-response method have led to ostensibly contradictory interpretations (e.g., Sanfey et al., 2003; Knoch et al., 2006; Tabibnia et al., 2008).
