# Supplementary material for: Short- and long-run goals in ultimatum bargaining: impatience predicts spite-based behavior
Source: Front Behav Neurosci. 2015 Aug 17;9:214. doi: 10.3389/fnbeh.2015.00214 (PMC4538919; doi:10.3389/fnbeh.2015.00214)
Supplement: Supplementary file 1 [file Supporting_Information.PDF]

Supporting Information (SI) for

Short- and long-run goals in ultimatum bargaining: impatience predicts spite-based behavior

Antonio M. Espín, Filippos Exadaktylos, Benedikt Herrmann & Pablo Brañas-Garza

S1. Materials and Methods

The survey-experiment was conducted from November 23rd to December 15th 2010, recruiting a total of 835 individuals from the adult population of the city of Granada, Spain. The sample was representative in terms of the geographical location of households within the city, age and gender of the participants (detailed information regarding sampling procedures as well as experimental protocols and games' instructions can be found in Exadaktylos et al. [S1]). Participants were ex-ante informed that the data would be used for scientific purposes only, that responses would never be linked to the identity of the respondent and that the procedures followed for the study and data acquisition were in accordance to the Spanish law on data protection guaranteeing full anonymity. Each respondent was interviewed in his/her own house by a pair of interviewers (108 pairs in total). Interviewers were last-year university students from the University of Granada, enrolled on a course on "field experiments", which involved their training on the methodology and conduction of survey-experiments. Their performance was carefully monitored by the main researchers through a web-based system and linked to their final grade in the course.

For statistical analysis purposes, we have used only those participants whose responses in both the delay discounting task and the ultimatum game were complete and reliable. This involved excluding those individuals making multiple switching or following

non-monotonic patterns in either the delay discounting task or the Ultimatum Game, and those never switching from the sooner to the later reward in any of the two DD tasks (see below). Additionally, only complete observations with regards to control variables were included, which is the usual practice when dealing with field data. The final sample consists of 713 complete observations (average age 36.7±16.6 (SD), 54.14% female).

S1.1. The delay discounting task

Two series of intertemporal decisions involving hypothetical monetary rewards were presented. One of the interviewers read each of the decisions involved in both subtasks, one at a time, and the interviewee gave his or her responses verbally. Participants faced a total of six decisions in each task. In the first decision of the short-run DD subtask, participants had to choose between €5 to be received "today" (sooner option) and €5 to be received "tomorrow" (later option). The remaining five decisions kept the sooner reward constant while increasing the later reward, in the following order: €6, €7, €8, €9, €10. In the first decision of the long-run DD subtask, participants had to choose between €150 to be received in one month time (sooner option) and €150 to be received in seven months time (later option). The remaining five decisions kept the sooner reward constant while increasing the later reward, in the following order: €170, €190, €210, €230, €250.

Figure S1. Distribution of subjects' choices in the DD tasks

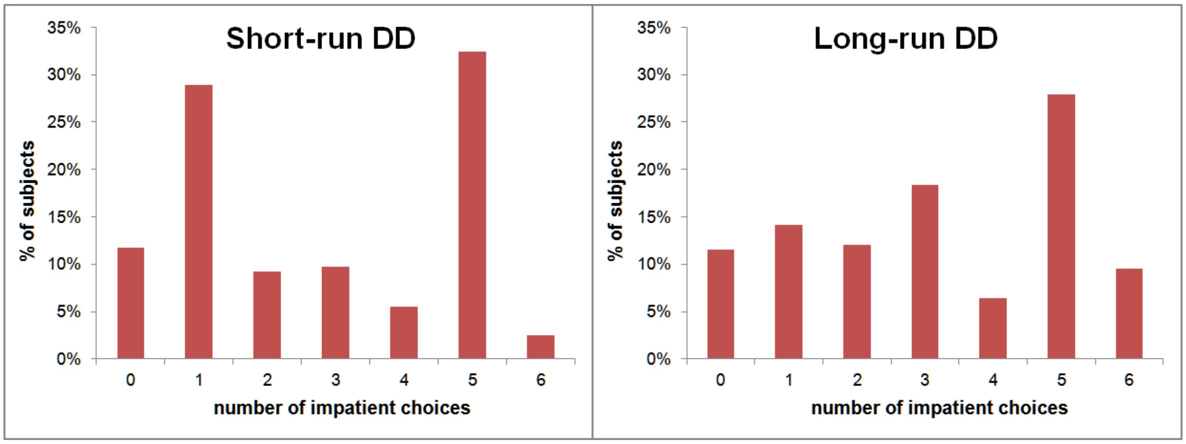

This task was specifically adopted to meet the particular purposes and restrictions of the survey-experiment. The two discounting measures captured by the task were not designed in order to allow for parameterization (i.e., in order to estimate the individuals'

discount functions in a particular functional form) but rather in order to allow a non-parametric characterization, that is, the number of impatient responses. The distribution of subjects' choices in the task is shown in Figure S1. In the case of the short-run DD, two categories concentrate more than 60% of subjects, while choices in the long-run DD are more uniformly distributed.

In the literature on DD, there is a long-lasting discussion regarding which particular functional form best characterizes individuals' discounting. While some scholars suggest that discount rates are constant (i.e., exponential, time-consistent discounting) others argue that discount rates decline over time (i.e., a (quasi-) hyperbolic functional form, which involves time-inconsistent preferences; see [S2, S3]). Due to the restrictions imposed by the nature of our survey-experiment, however, the discounting elicitation task was not designed to allow us estimate the parameters of the individuals' discount functions. Consequently, an in-depth analysis of the prevalence of constant versus declining discount rates cannot be performed.

According to dual-valuation neurobiological theories of DD [S4, S5], decisions involving immediate and delayed rewards are evaluated in the brain differently than those involving only delayed rewards. Concretely, the so-called "beta" and "delta" systems would be at work for valuating delayed rewards [S6]. The beta system refers to short-term discounting. It is supposed to steeply discount all non-immediate rewards by a fixed proportion β (there is an immediacy premium or a present bias). The more patient delta system captures long-term discounting and is supposed to discount delays in a less-steep, exponential manner. That is, for each unit of time that constitutes the delay to delivery, the value of a reward is discounted by δ. Accordingly, the subjective value of a delayed reward would result from subtracting the non-immediacy penalization (beta system) from the discounted value of the reward (delta system). In particular, the beta-delta model formalizes the individual's discount function as $V_d = \beta\delta^t V_u$, where V_d is the discounted psychological value of a reward with (undiscounted) value V_u, which will be received in t time units. β and $\delta \in (0, 1]$ are the "beta" and "delta" discount factors, respectively. The higher these discount factors are, the more patient is the individual, as delayed rewards are valued more (i.e. they are discounted less). Thus, in our long-run subtask, it is straightforward to see that

the beta (present-bias) component has no influence on decision making. For instance, imagine that an individual is indifferent between 150€ in one month (30 days) and 170€ in seven months (210 days) (2nd decision of the subtask). Indifference implies that the discounted values of both options are identical. Thus, assuming that utility is linear over the relevant range [S7], $\beta\delta^{30}$*150= $\beta\delta^{210}$*170, or δ^{180}=150/170. We see that beta is cancelled out from the equation, so present bias does not influence decisions in the long-run DD subtask. However, the beta system should have an influence in the short-run DD subtask. For instance, for an individual who is indifferent between 5€ "today" and 6€ "tomorrow" (2nd decision of the subtask), we obtain 5= $\beta\delta$*6. Indeed, here we see that both beta and delta parameters are present in the reduced equation. Note that even if the relatively small number of decisions in our DD task does not allow a very precise estimation of these parameters (see footnote 1), the existence of these two different processes could entail that the two measures we obtain are associated with different behavioral patterns in the UG. For instance, if we find that the short-run DD measure is related to UG behavior but the long-run DD is not, it could be that it is the beta but not the delta system which influences decision making in the UG.

On the contrary, single-valuation theories [S8] argue in favor of a single psychological process evaluating both immediate and delayed rewards. This process would discount the value of all rewards in function of the delay to their delivery, following a hyperbolic form [S9]. According to this view, no behavioral differences should be found for the two DD measures we gathered.

S1.2. The ultimatum game

All participants made decisions in both roles of the game. As proposers, subjects made an offer to a randomly matched participant on how to split a pie of €20 (in €2 increments). The average offer was 46.2% (±0.7; robust SEM clustered by interviewers) of the pie. In the role of responders, subjects had to state their willingness to accept or reject each of the following proposals (proposer's payoff (€), responder's payoff (€)): (20, 0); (18, 2); (16, 4); (14, 6); (12, 8); (10, 10). With this method we obtained the minimum acceptable offer (MAO) of each subject. The average MAO was 35.0% (±0.9) of the pie. These averages are within the range found in previous ultimatum field experiments [S10]. Figure

S2 displays the distribution of offers and MAOs (expressed as fraction of the pie) in the sample.

The instructions of the game were read aloud by one of the interviewers. Subsequently, participants were required to privately write down their choices on a decision sheet, which was later introduced in an envelope to ensure a double-blind procedure. The order in which decisions were made was randomized across participants. Matching and payment took place within the next two weeks and the average earnings among winners were €9.60.

Figure S2. Distribution of subjects' choices in the UG

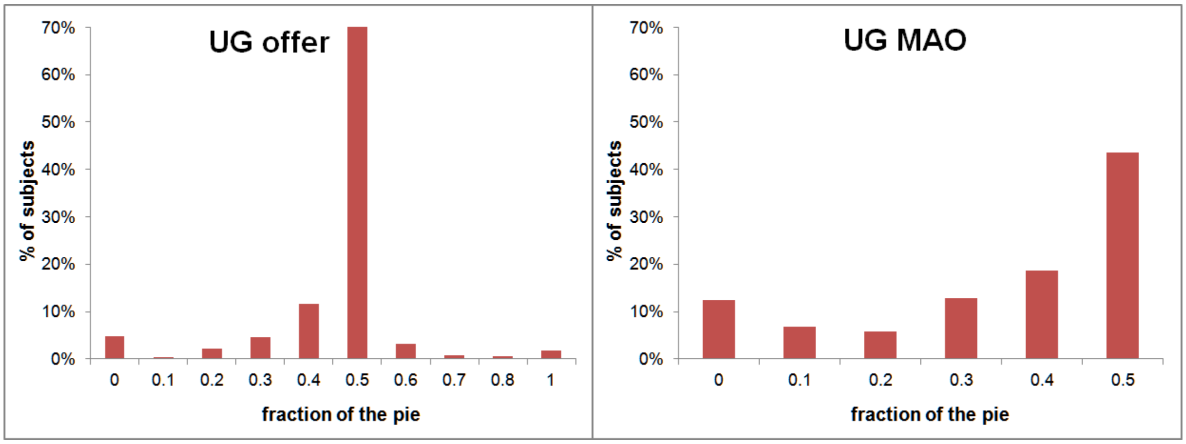

S1.3. Calculation of expected payoffs

To calculate the expected payoffs of participants we simulated a perfect random matching. In particular, the UG offer (o_i) and the MAO (m_i) of every subject was matched with, respectively, the MAO (m_j, j=1…N, j≠i) and the offer (o_j, j=1…N, j≠i) of every other subject. After interacting with all the other subjects in both roles of the game, each subject's *own payoff* will be simply given by her mean payoff across the 712 encounters. Furthermore, for every subject we have also computed the *other's payoff*, which is given by the mean payoff of the other 712 participants from their interactions with this subject (again, taking both roles into account). That is, the *other's payoff* of participant i is a measure of how much one expects to earn when interacting with i. This measure is not to be confounded with the mean *own payoff* of the other subjects.

The average payoff for individual i after interacting in both roles with individual j is given by [S11, S12]:

a) ½(1-o_i+o_j) if $o_i \geq m_j$ and $o_j \geq m_i$;
b) ½(1-o_i) if $o_i \geq m_j$ and $o_j < m_i$;
c) ½o_j if $o_i < m_j$ and $o_j \geq m_i$;
d) 0 if $o_i < m_j$ and $o_j < m_i$.

Then, the expected "*own payoff*" for individual i is calculated by weighting each of these four possible payoffs by the probability of that specific case occurring in the sample (i.e., its relative frequency). Likewise, the average payoff for individual i's partner (i.e., individual j; the "other") is given by:

a) ½(1-o_j+o_i) if if $o_i \geq m_j$ and $o_j \geq m_i$
b) ½o_i if $o_i \geq m_j$ and $o_j < m_i$;
c) ½(1-o_j) if $o_i < m_j$ and $o_j \geq m_i$;
d) 0 if $o_i < m_j$ and $o_j < m_i$.

Again, the expected "*other's payoff*" for individual i is calculated by assigning weights (same as above) to the latter payoffs. Let us illustrate how we calculate both *own payoff* and *other's payoff* with an example.

We use a simple case with only three subjects whose strategies are (o_1, m_1) = (8, 4), (o_2, m_2) = (2, 6) and (o_3, m_3) = (4, 10). We look into the case of subject 1. Performing the random matching, this subject is matched with all other subjects; subjects 2 and 3 in this example. From her interaction with subject 2, subject 1's payoff is given by ½(20-8)=6 since 8≥6 and 2<4 (case b) above). Similarly, interacting with subject 3, subject 1's payoff will be: ½4=2 since 8<10 and 4≥4 (case c) above). To calculate the *own payoff* of subject 1, we attach the appropriate weight to the above payoffs, which in this case is 0.5 for both payoffs (i.e., their relative frequency is 50%; the weight of the remaining possible cases is obviously zero). Thus the *own payoff* is 0.5*6 + 0.5*2 = 4.

Let us now calculate the *other's payoff* corresponding to subject 1. From her interaction with subject 1, subject 2's payoff will be determined as follows: ½8=4 since 8≥6 and 2<4 (case b) above). From her interaction with subject 1, subject 3's payoff will

be: ½(20-4)=8 since 8<10 and 4≥4 (case c) above). In order to calculate the *other's payoff* we attach the corresponding weight to each payoff, which again in this particular example is 0.5 in both cases. Thus the *other's payoff* for subject 1 is 0.5*4 + 0.5*8 = 6.

Similarly we can calculate the *own payoff* of subjects 2 and 3, which are 2 and 4 respectively. It is important to note that the *other's payoff* of subject 1 is different than the mean *own payoff* of the other two subjects, which is (2+4)/2 = 3.

S2. Supporting analyses

S2.1. Variables description

In the next subsection, we will provide the estimates of the regressions summarized in Table 1 of the main text. "Short-run DD" and "long-run DD" refer to the number of impatient responses the individual made for each delay; "combined DD" is the average of the two (these three categorizations of DD are normalized to the interval [0, 1]); "highDD vs. lowDD" takes the value 1 if the individual belongs to the top 33% and 0 if belongs to the bottom 33% of the distribution of "combined DD" (observations falling in the central 33% are missing in the analyses using this variable).

The control variables employed in the regressions are:

- *Age* ϵ [16, 89].

- *Male*: 1 if male, 0 if female.

- *Married*: 1 if married, 0 otherwise.

- *House inc* ϵ [0, 4500]: average household monthly income in the last year (in €500 increments).

- *Educ level* ϵ [0, 8]: no studies (0), incomplete primary school (1), complete primary school (2), incomplete secondary school (3), complete secondary school (4), incomplete university diploma or technical degree (5), complete university diploma or technical degree (6), incomplete bachelor or postgraduate degree (7), complete bachelor or postgraduate degree (8).

- *Cognit ab* ∊ [0, 5]: number of correct answers to the following five mathematical questions:

1. *If the probability of being infected by an illness is 10%, how many persons of a group of 1000 would be infected by that kind of illness?*

2. *If there are 5 persons that own the winning lottery ticket and the prize to be shared is two million euros, how much money would each person receive?*

3. *Suppose that you have 100€ in a savings account and the rate of interest that you earn from the savings is 2% per year. If you keep the money in the account for 5 years, how much money would you have at the end of these 5 years?:*
a. More than 102€
b. 102€ exactly
c. Less than 102€
d. S/he cannot/do not want to answer

4. *Suppose that you have 100€ in a savings account. The account accumulates a 10% rate of interest per year. How much money would you have in your account after two years?*

5. *The total cost of a bat and a ball is 1.10 euros. The bat costs 1 euro more than the ball. How many cents does the ball cost?*

- *Risk 1*: 1 if option b, 0 if option a in the question:

We flip a coin. Choose one of the following options:
a. Take 1.000 Euros no matter if it is heads or tails.
b. Take 2.000 Euros if it is heads and nothing if it is tails.

- *Risk 2*: 1 if option a, 0 if option b in the question:

Choose one of the following options:
a. Take a lottery ticket with 80% chance of winning 45 Euros and 20% chance of winning nothing.
b. Take 30 Euros.

- *Risk 3*: 1 if ‘Yes’, 0 if ‘No’ in the question:

> *Would you accept the following deal? We flip a coin. If it is heads you win 1,500 Euros and if it is tails you lose 1,000 Euros: Yes (Y), No (N)*

S2.2. Regression analyses

Tables S1 to S4, present the OLS regressions using “short-run DD”, “long-run DD”, “combined DD”, and “highDD vs. lowDD”, respectively, to characterize delay discounting as explanatory variables. The dependent variables (expressed as a fraction of the pie) in columns (1) to (5) are, respectively: offer, MAO, offer-MAO, *own payoff*, and *other’s payoff*. In all regressions we control also for order effects. Robust standard errors clustered by interviewers are presented in brackets.

From the regression analyses it appears that the effect of DD on behavior and payoffs does not crucially depend on which period-length is used to measure it, though it is more prominent in the case of the longer delay. It might be that the “beta” component of DD (see Text S1.1) has no influence on behavior and it is the “delta” component that drives the results. This would explain why the long-run DD yields better estimates. However, this result can also be due to the higher noise of observations in the short-run DD subtask (see Figure S1). In any case, we can discard that it is *only* the beta system that relates to UG behavior.[1]

Among the control variables, only the subjects’ cognitive abilities, marital status and risk preferences yield significant coefficients across models. Cognitive abilities relate positively to MAO and *own payoff*, as reported in the main text. Different risk-taking measures report different relationships with behavior, thus making an interpretation difficult (also, a high level of collinearity between the measures could influence the estimation). Finally, married subjects are found to be less willing to reject low offers (i.e., they have a lower MAO), although weakly.

[1] Although the task was not designed to allow to accurately estimate individuals’ discount functions, we have used the data to analyze the separate effects of empirically estimated “beta” and “delta” discount factors (following the methodology employed in Burks et al. [S7]) and, as expected, delta predicts subjects’ behavior slightly better than beta but still both measures are linked to the same patterns (available upon request from the authors).

Table S1. UG behavior and expected payoffs as a function of *short-run DD*

	offer	MAO	offer-MAO	*own payoff*	*other's payoff*
	(1)	(2)	(3)	(4)	(5)
short-run DD	-0.0294	0.0428*	-0.0722**	-0.0115	-0.0252*
	(0.021)	(0.022)	(0.032)	(0.007)	(0.013)
age	0.0003	0.0008	-0.0005	0.0000	0.0003
	(0.000)	(0.001)	(0.001)	(0.000)	(0.000)
male	-0.0025	-0.013	0.0104	-0.0043	0.0005
	(0.010)	(0.013)	(0.015)	(0.004)	(0.006)
married	-0.0070	-0.0325*	0.0255	0.0053	-0.0038
	(0.014)	(0.017)	(0.024)	(0.005)	(0.008)
house inc	-0.0000	0.0000	-0.0000	-0.0000	-0.0000
	(0.000)	(0.000)	(0.000)	(0.000)	(0.000)
educ level	0.0021	-0.0041	0.0062	0.0011	0.0027
	(0.004)	(0.003)	(0.005)	(0.001)	(0.002)
cognit ab	0.0014	0.0117**	-0.0102	0.0052***	-0.0014
	(0.005)	(0.005)	(0.007)	(0.002)	(0.003)
risk 1	-0.0110	0.0560***	-0.0670**	-0.0044	-0.0133
	(0.016)	(0.0189)	(0.026)	(0.006)	(0.010)
risk 2	-0.0070	-0.0153	0.0083	-0.0021	-0.0008
	(0.013)	(0.017)	(0.023)	(0.005)	(0.009)
risk 3	0.0422**	-0.0381	0.0803*	0.0001	0.0266*
	(0.020)	(0.031)	(0.041)	(0.009)	(0.014)
constant	0.4581***	0.2628***	0.1954***	0.4475***	0.4398***
	(0.036)	(0.050)	(0.070)	(0.013)	(0.023)
R^2	0.0415	0.0655	0.0511	0.0635	0.0523
F	2.42***	2.15***	1.72**	3.78***	1.47*
obs.	713	713	713	713	713

Notes: OLS estimates. Robust standard errors clustered by interviewers in brackets.*, **, *** indicate significance at the 0.10, 0.05 and 0.01 levels, respectively. All regressions control for order effects.

Table S2. UG behavior and expected payoffs as a function of *long-run DD*

	**offer**	**MAO**	**offer-MAO**	***own payoff***	***other's payoff***
	**(1)**	**(2)**	**(3)**	**(4)**	**(5)**
long-run DD	-0.0324**	0.0393*	-0.0717***	0.0018	-0.0269***
	(0.016)	(0.020)	(0.024)	(0.006)	(0.010)
age	0.0002	0.0008	-0.0006	0.0000	0.0003
	(0.000)	(0.000)	(0.001)	(0.000)	(0.000)
male	-0.0018	-0.0138	0.0120	-0.0041	0.0011
	(0.010)	(0.012)	(0.015)	(0.004)	(0.006)
married	-0.0059	-0.0334*	0.0274	0.0046	-0.0030
	(0.014)	(0.018)	(0.025)	(0.005)	(0.009)
house inc	-0.0000	0.0000	-0.0000	-0.0000	-0.0000
	(0.000)	(0.000)	(0.000)	(0.000)	(0.000)
educ level	0.0023	-0.0044	0.0067	0.0012	0.0029
	(0.004)	(0.003)	(0.005)	(0.001)	(0.002)
cognit ab	0.0012	0.0120**	-0.0108	0.0051***	-0.0016
	(0.005)	(0.005)	(0.007)	(0.002)	(0.003)
risk 1	-0.0120	0.0569***	-0.0688***	-0.0036	-0.0141
	(0.016)	(0.019)	(0.025)	(0.006)	(0.010)
risk 2	-0.0049	-0.0182	0.0133	-0.0015	0.0010
	(0.013)	(0.017)	(0.023)	(0.005)	(0.008)
risk 3	0.0407**	-0.0364	0.0770*	0.0004	0.0254*
	(0.020)	(0.031)	(0.040)	(0.009)	(0.014)
constant	0.4659***	0.2564***	0.2095***	0.4415***	0.4459***
	(0.039)	(0.052)	(0.074)	(0.013)	(0.025)
R^2	0.0423	0.0646	0.0509	0.0601	0.0532
F	2.17***	2.14***	1.92**	4.37***	1.57*
obs.	713	713	713	713	713

Notes: OLS estimates. Robust standard errors clustered by interviewers in brackets.*, **, *** indicate significance at the 0.10, 0.05 and 0.01 levels, respectively. All regressions control for order effects.

Table S3. UG behavior and expected payoffs as a function of *combined DD*

	offer	MAO	offer-MAO	*own payoff*	*other's payoff*
	(1)	(2)	(3)	(4)	(5)
combined DD	-0.0437**	0.0581**	-0.1018***	-0.0070	-0.0369***
	(0.021)	(0.024)	(0.032)	(0.008)	(0.013)
age	0.0002	0.0008	-0.0006	0.0000	0.0003
	(0.000)	(0.001)	(0.001)	(0.000)	(0.000)
male	-0.0021	-0.0133	0.0112	-0.0041	0.0008
	(0.010)	(0.012)	(0.015)	(0.004)	(0.006)
married	-0.0054	-0.0343*	0.0289	0.0052	-0.0026
	(0.014)	(0.017)	(0.024)	(0.005)	(0.009)
house inc	-0.0000	0.0000	-0.0000	-0.0000	-0.0000
	(0.000)	(0.000)	(0.000)	(0.000)	(0.000)
educ level	0.0022	-0.0042	0.0064	0.0012	0.0027
	(0.004)	(0.003)	(0.005)	(0.001)	(0.002)
cognit ab	0.0014	0.0117**	-0.0103	0.0051***	-0.0014
	(0.005)	(0.005)	(0.007)	(0.002)	(0.003)
risk 1	-0.0126	0.0580***	-0.0707***	-0.0043	-0.0147
	(0.016)	(0.019)	(0.026)	(0.006)	(0.010)
risk 2	-0.0063	-0.0164	0.0101	-0.0016	-0.0002
	(0.013)	(0.017)	(0.023)	(0.005)	(0.008)
risk 3	0.0408**	-0.0363	0.0771*	0.0000	0.0255*
	(0.020)	(0.031)	(0.040)	(0.009)	(0.014)
constant	0.4707***	0.2477***	0.2230***	0.4466***	0.4502***
	(0.038)	(0.050)	(0.071)	(0.013)	(0.024)
R^2	0.0443	0.0680	0.0560	0.0610	0.0567
F	2.39***	2.24***	1.97***	4.30***	1.59*
obs.	713	713	713	713	713

Notes: OLS estimates. Robust standard errors clustered by interviewers in brackets.*, **, *** indicate significance at the 0.10, 0.05 and 0.01 levels, respectively. All regressions control for order effects.

Table S4. UG behavior and expected payoffs as a function of *highDD vs. lowDD*

	**offer**	**MAO**	**offer-MAO**	***own payoff***	***other's payoff***
	**(1)**	**(2)**	**(3)**	**(4)**	**(5)**
hDD vs. lDD	-0.0281*	0.0431***	-0.0711***	-0.0069	-0.0231**
	(0.014)	(0.015)	(0.021)	(0.005)	(0.009)
age	-0.0003	0.0007	-0.0011	-0.0002	0.0000
	(0.001)	(0.001)	(0.001)	(0.000)	(0.000)
male	-0.0016	-0.0118	0.0102	-0.0062	0.0017
	(0.016)	(0.014)	(0.022)	(0.005)	(0.010)
married	0.0022	-0.0401*	0.0423	0.0088	0.0011
	(0.017)	(0.021)	(0.029)	(0.007)	(0.011)
house inc	-0.0000	0.0000	-0.0000	-0.0000	-0.0000
	(0.000)	(0.000)	(0.000)	(0.000)	(0.000)
educ level	0.0056	-0.0086**	0.0143*	0.0009	0.0048
	(0.005)	(0.004)	(0.007)	(0.002)	(0.003)
cognit ab	0.0022	0.0167**	-0.0145	0.0063**	-0.0014
	(0.007)	(0.006)	(0.010)	(0.003)	(0.004)
risk 1	-0.0147	0.0613**	-0.0761**	0.0001	-0.0163
	(0.020)	(0.026)	(0.035)	(0.008)	(0.014)
risk 2	-0.0096	-0.0078	-0.0018	-0.0093	-0.0036
	(0.017)	(0.018)	(0.026)	(0.006)	(0.010)
risk 3	0.0399	-0.0434	0.0833	0.0012	0.0278
	(0.026)	(0.041)	(0.056)	(0.013)	(0.019)
constant	0.4342***	0.3606***	0.0736	0.4228***	0.3903***
	(0.042)	(0.051)	(0.079)	(0.023)	(0.036)
R^2	0.0611	0.0899	0.0774	0.0790	0.0711
F	2.47***	4.06***	2.09***	3.58***	1.71**
obs.	488	488	488	488	488

Notes: OLS estimates. Robust standard errors clustered by interviewers in brackets.*, **, *** indicate significance at the 0.10, 0.05 and 0.01 levels, respectively. All regressions control for order effects.

S2.3. Graphical representation of UG behavior and expected payoffs across DD

Figure S3

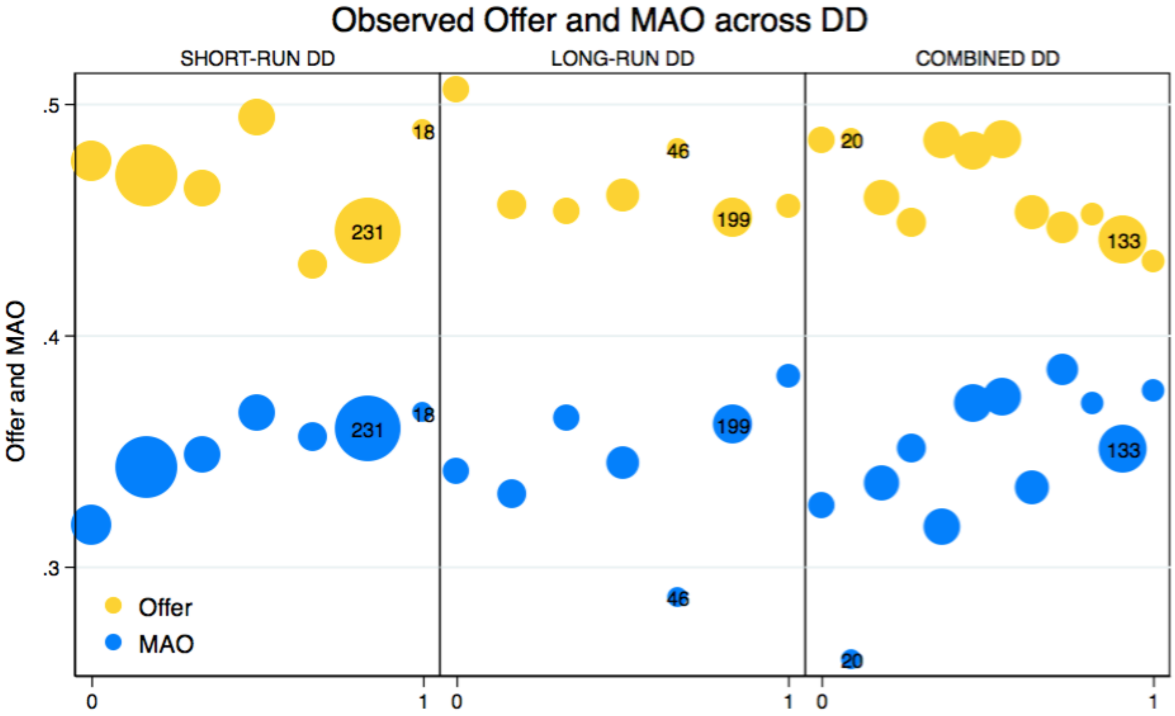

Figure S3. Observed mean Offer and MAO across short-run, long-run and combined DD. The size of the bubbles is proportional to the number of individuals in the corresponding category. The number of individuals is depicted for the largest and the smallest category.

Figure S4

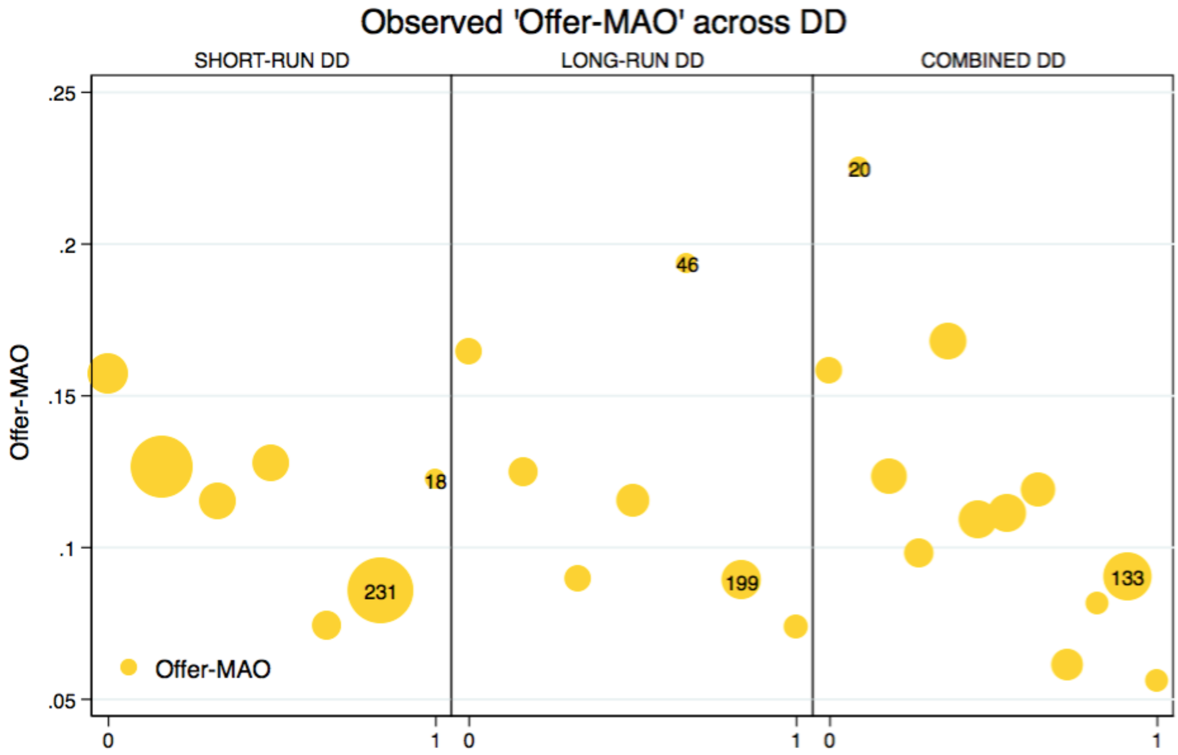

Figure S4. Mean ‘Offer-Mao’ across short-run, long-run and combined DD. The size of the bubbles is proportional to the number of individuals in the corresponding category. The number of individuals is depicted for the largest and the smallest category.

Figure S5

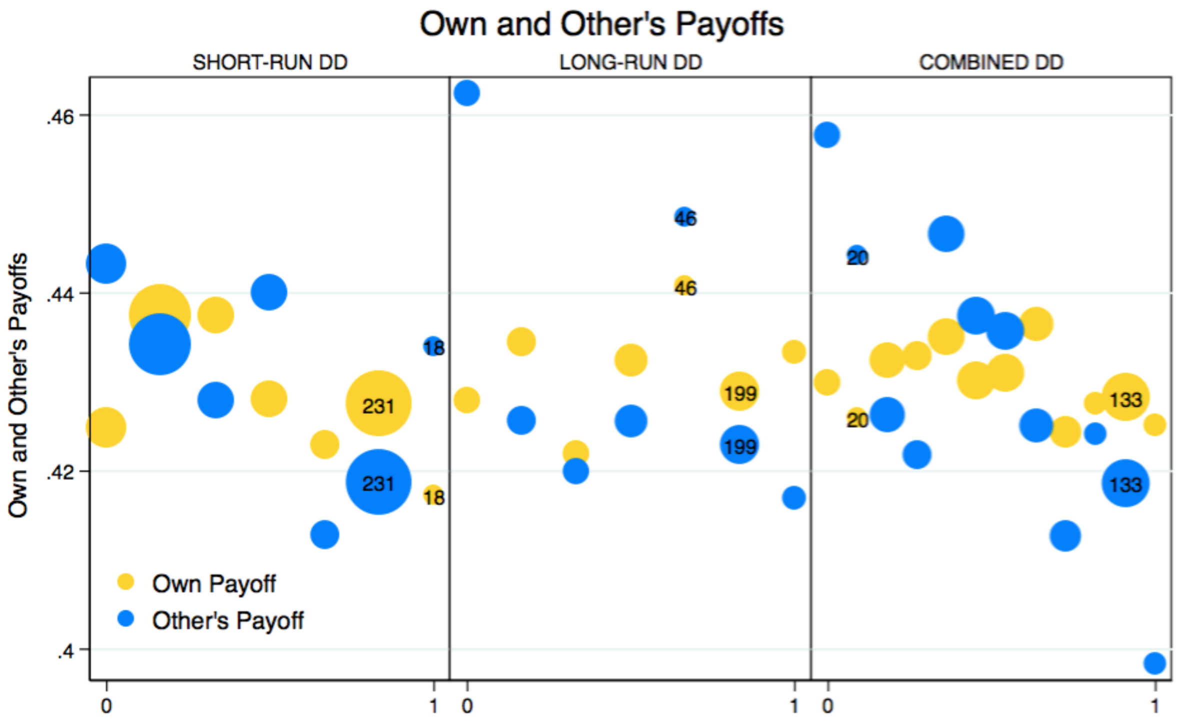

Figure S5. Own- and Other's Payoffs across long-run, short-run and combined DD. The size of the bubbles is proportional to the number of individuals in the corresponding category. The number of individuals is depicted for the largest and the smallest category.

S2.4. Further analyses

Figure S6

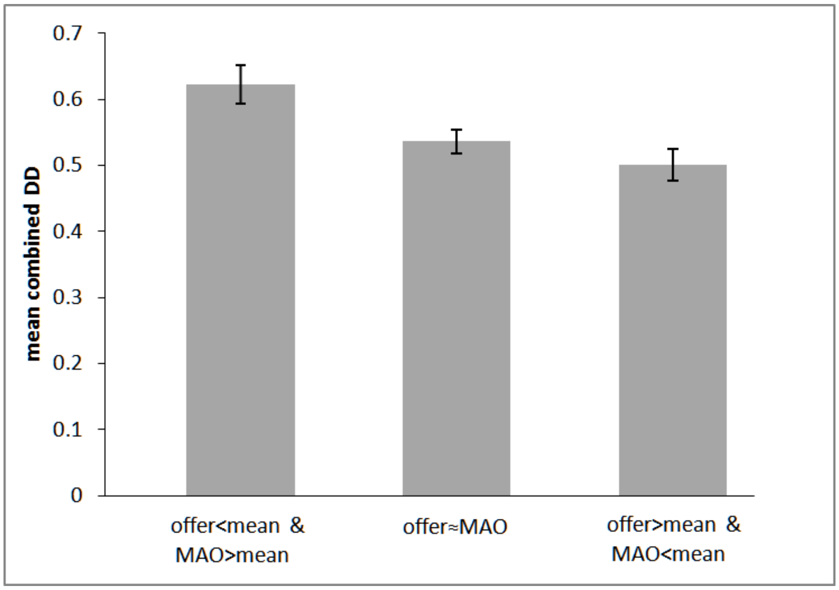

Figure S6. Mean impatience (± robust SEM) of individuals with: (i) offer<mean & MAO>mean (left bar) (n=90); (ii) either both offer & MAO>mean or both offer & MAO<mean (central bar) (n=433); (iii) offer>mean & MAO<mean (right bar) (n=190).

In Figure S6, we split the sample in three categories according to UG strategies and show the mean "combined DD" of participants within each category. The first category (left bar) refers to those participants offering less than the mean offer (46.2% of the pie) but whose MAOs exceed the mean MAO (35.0% of the pie). In the second category we include those participants whose offers and MAOs do not differ in their relation to the respective mean (i.e., both offer and MAO either below or above the mean). The last category (right bar) comprises those participants with offers above the mean offer but with MAOs below the mean MAO. It can be observed that the mean impatience decreases as we move from left to right, i.e. from the "most spiteful" to the "least spiteful" individuals. The combined DD of the left-hand group is significantly higher than that in each of the other two groups (ps<0.01, Mann-Whitney test, two-tailed), whereas it is higher in the middle group compared to the right-hand group, though not significantly so (p>0.16, Mann-Whitney test, two-tailed).

References

S1. Exadaktylos F, Espín AM, Brañas-Garza P (2013) Experimental subjects are not different. *Sci Rep* 3: 1213.

S2. Frederick S, Loewenstein G, O'Donoghue T (2002) Time discounting and time preference: a critical review. *J Econ Lit* 40: 351-401.

S3. Green L, Myerson J (2004) A discounting framework for choice with delayed and probabilistic rewards. *Psychol Bull* 130: 769-792.

S4. McClure SM, Laibson DI, Loewenstein G, Cohen JD (2004) Separate neural systems value immediate and delayed monetary rewards. *Science* 306: 503-507.

S5. McClure SM, Ericson KM, Laibson DI, Loewenstein G, Cohen JD (2007) Time discounting for primary rewards. *J Neurosci* 27: 5796-5804.

S6. Laibson D (1997) Golden eggs and hyperbolic discounting. *Q J Econ* 112: 443-478.

S7. Burks SV, Carpenter JP, Goette L, Rustichini A (2009) Cognitive skills affect economic preferences, strategic behavior, and job attachment. *Proc Natl Acad Sci USA* 106: 7745-7750.

S8. Kable JW, Glimcher PW (2007) The neural correlates of subjective value during intertemporal choice. *Nat Neurosci* 10: 1625-1633.

S9. Ainslie GW (1992) *Picoeconomics: The Strategic Interaction of Successive Motivational States Within the Person* (Cambridge University Press, Cambridge).

S10. Henrich J et al. (2006) Costly punishment across human societies. *Science* 312: 1767-1770.

S11. Rand DG, Tarnita CE, Ohtsuki H, Nowak MA (2013) Evolution of fairness in the one-shot anonymous Ultimatum Game. *Proc Natl Acad Sci USA* 110: 2581-2586.

S12. Iranzo J, Floría LM, Moreno Y, Sánchez A (2012) Empathy emerges spontaneously in the ultimatum game: small groups and networks. *PloS One* 7: e43781.
